# Surface expression and genotypes of Toll-*like* receptors 2 and 4 in patients with juvenile idiopathic arthritis and systemic lupus erythematosus

**DOI:** 10.1186/1546-0096-11-9

**Published:** 2013-03-05

**Authors:** Martina Kirchner, Anja Sonnenschein, Simon Schoofs, Peter Schmidtke, Volker N Umlauf, Wilma Mannhardt-Laakmann

**Affiliations:** 1 University Hospital of Mainz, Department of Pediatrics, Division of Pediatric Immunology and Rheumatology, Langenbeckstrasse 1, Mainz, D-55131, Germany; 2 University Hospital of Aachen, Department of Pediatrics, Pauwelstrasse 30, Aachen, D-52070, Germany

**Keywords:** Juvenile idiopathic arthritis, Pathogenesis, Innate immunity, Toll-*like* receptors

## Abstract

**Background:**

Chronic arthritis is a common feature of juvenile idiopathic arthritis (JIA) and systemic lupus erythematosus (SLE). It was subsequently discovered that Toll-*like* receptors (TLRs) are able to upregulate cytokine production in response to endogenous ligands released after tissue damage, suggesting that TLRs can maintain an inflammatory response even in absence of pathogen. Thus, TLRs may contribute to increased inflammation in JIA and SLE patients. The aim of this study was to investigate the role of TLRs in JIA and SLE. We examined the in vivo expression and polymorphisms of TLR2 and TLR4 in peripheral monocytes of patients with JIA and SLE during active and inactive disease phases.

**Methods:**

This single center cohort study consisted of JIA and SLE affected children and control subjects. TLR2 and TLR4 protein expression on CD14^+^ monocytes was examined by flow cytometry. TLR2 and TLR4 genotypes were determined using the polymerase chain reaction-restriction fragment length polymorphism method (RFLP-PCR).

**Results:**

A significant reduction in the level of TLR4 expression (p ≤ 0.001) was observed on monocytes of patients with JIA and SLE compared with that of healthy control subjects. There was no correlation between the TLR2 or TLR4 genotypes and the observed differential TLR protein expression on monocytes.

**Conclusions:**

To conclude, our observations suggest involvement of investigated TLRs in the pathogenesis of JIA and SLE. It still remains to be elucidated whether reduced TLR4 expression is cause of chronic arthritis or a result of some feedback loop.

## Background

JIA refers to a group of chronic childhood arthropathies of unknown aetiology. SLE is the prototypic systemic autoimmune disease and is characterized by B cell hyperreactivity and the production of autoantibodies [1]. Chronic arthritis, a common feature of JIA and SLE, in the past was mainly attributed to dysregulated tolerance mechanisms of the adaptive immune system, particularly T-cells. However, the innate immune system has an important role in the pathogenesis of arthritis. Toll-*like* receptors (TLRs) are a key link between infection, injury and inflammation [2]. They recognize pathogen- and danger-associated molecular patterns (PAMPs and DAMPs), and subsequently trigger a pro-inflammatory cascade, which induces the transcription of multiple proinflammatory cytokine genes as well as dendritic cell maturation [3,4]. Cell wall components of various Gram-positive and Gram-negative pathogens, stress proteins and cell decomposition products are predominantly identified by the Toll-*like* receptors 2 and 4 [3,5]. Although TLR-mediated inflammation is an important aspect of host defense, it is also linked to pathogenesis of several autoimmune diseases, like rheumatoid arthritis (RA) and SLE [4,6-9]. Feedback loops between DAMPS, PAMPs and their overlapping receptors may link infectious triggers and disease flares [10]. Self-molecules, (e.g. S100 proteins) indicating synovial tissue damage amplify inflammatory arthritis [11]. Serum concentrations of S100A8/S100A9 proteins correlate well with the disease activity in children [12]. Some studies suggest that patients with clinically inactive JIA, but elevated levels of S100A proteins, may be at risk for disease flares [13]. Children with systemic onset of JIA show up to 20-fold higher S100A serum protein concentrations than those found in other inflammatory disorders. In SLE elevated serum concentrations also correlate significantly with disease activity index [14]. The elevated expression levels of Toll-*like* receptors 2, TLR3 and TLR4 have already been associated with the chronic joint inflammation in adult RA patients [15,16].

Associations between different polymorphisms within the TLR2 and TLR4 genes or within the promoter region of the TLR9 gene and susceptibility for RA and SLE are subjects of great controversy [17-19]. Few studies exist to date on the importance of innate immunity and of, in particular, Toll-*like* receptors in the pathogenesis of JIA and SLE. Recently, two common cosegregating missense mutations, Asp299Gly and Thr399Ile, affecting the extracellular domain of the TLR4 protein, have been characterized [20]. Both mutations lead to an attenuated efficacy of lipopolysaccharide signaling and a reduced capacity to elicit inflammation. Also for the TLR2 gene two SNPs (Arg677Trp and Arg753Gln) have been identified that abrogate the ability of TLR2 to mediate a response to bacterial cell wall components [21].

DAMPS are found in high concentrations in inflamed tissue, where neutrophils and monocytes belong to the most abundant cell types. We hypothesized that the enduring local inflammation triggered by DAMPS may correlate with levels of TLR protein expression on CD14^+^ monocytes. We therefore decided to investigate the TLR2 and TLR4 protein expression on CD14^+^ monocytes of JIA and pediatric SLE patients during active and inactive disease phases as well as the TLR expression levels of healthy individuals. Furthermore we hypothesized that the two single nucleotide polymorphisms (SNPs) of TLR2-gene (Arg677Trp and Arg753Gln) and TLR4-gene (Asp299Gly and Thr399Ile) are implicated in the pathogenesis of JIA and SLE. The current study compared the allele frequency and genotype distribution for these mutations among JIA respectively SLE patients and healthy control subjects.

## Methods

### Patients and samples

This single center cohort study consisted of JIA and SLE affected children and control subjects and was conducted in the period 2008–2010. All JIA patients fit the ILAR classification criteria for childhood arthritis and provided written informed consent before enrolment. SLE patients who satisfied ECLAM criteria were enrolled in the study. Peripheral venous blood was drawn from 76 patients of our pediatric rheumatological outpatient clinic and from 67 similar gender non-related young adults to serve as healthy controls. The study protocol was approved by the institutional ethics committee (#837.169.08).

### Patient demographics

Sixty-five JIA patients were enrolled after consent: 19 patients with systemic JIA (3 children under flare), 28 patients with oligoarthritis (3 children under flare), 9 patients with seronegative polyarthritis (3 children under flare) and 11 patients with enthesitis related arthritis (3 children under flare). Furthermore we enrolled 9 SLE patients (1 patient under flare). Comprehensive clinical information was collected at each JIA patient visit, including history, physical examination (including presence of fever, rash and joint count), and clinical laboratory values [erythrocyte sedimentation rate (ESR), and C-reactive protein (CRP)]. Clinical status at each visit was graded according to a scoring system developed by our group to grade severity of systemic disease manifestations or arthritis. Each sample was classified as “flare” (active disease; score of 5 or above) or “quiescence” (inactive disease; score of 0). All patients were under antiphlogistic (Naproxene) and/or immunosuppressive (Methotrexate, Cyclosporin A, Azathioprin, steroids) therapy, dependent upon their JIA subtype. Characteristics of the study subjects are shown in Table 1.

**Table 1:** Patient demographics

	**Flow cytometry (TLR)**	**RFLP (TLR)**
Number of samples analyzed patients/controls	76:67	76:67
Age at enrollment	13.4 (0–26)	13.4 (0–26)
[median (range) years]		
Age at onset	6.3 (0–12)	6.3 (0–12)
[median (range) years]		
Disease duration	4.7 (1–11)	4.7 (1–11)
[median (range) years]		
Disease activity		
No or low activity [n]	63	63
High activity/flare [n]	13	13
ESR (mm/h)	19 (0–103)	19 (0–103)
CRP (mg/l)	15 (1–372)	15 (1–372)
Male/female	26:50	26:50
HLA B27 positive	11	11
Patients on		
DMARDS	29	29
NSAIDS	12	12
Steroids	3	3

### Flow cytometry for detection of TLR2- and TLR4-surface expression

Two-colour flow cytometry was applied to freshly isolated whole blood samples, to investigate the expression levels of TLR2 and TLR4 on peripheral blood monocytes of JIA and SLE patients and healthy control subjects. For surface staining 100 μl of whole blood samples were incubated with the following anti-human primary antibodies for 15 minutes in the dark at 4°C: anti-CD14 monoclonal antibody (mAb; 10 μl per 100 μl blood, FITC or PE labelled, clone 8 G3; Diaclone, Besançon, France), anti-TLR2 mAb (10 μl, FITC labelled, clone HTA 125; eBioscience, San Diego, CA) or TLR4 mAb (10 μl, PE labelled, clone HTA 125; eBioscience, San Diego, CA). Isotype-matched antibody controls were used to detect non-specific staining (Mouse IgG2a Isotype Control, FITC or PE labelled; eBioscience, San Diego, CA). Subsequently, red blood cells were lysed with lysing solution (FACS; BD Biosciences) and removed by washing two times with cell wash solution (FACS; BD Biosciences). The immunostained cells were fixed with 0.5% paraformaldehyde. Monocytes were identified and gated according to their characteristic forward- and side-scatter flow cytometry profiles and their expression of CD14. A six-parameter flow cytometer (FACSCalibur, Becton Dickinson) was used for data acquisition. Routinely, 8.0 x 10^3^ CD14^+^ cells were counted. Flow cytometry data were analyzed by using CellQuest software (version 5.3 for Macintosh; BD Bioscience Immunocytometry Systems, San Jose, CA). Mean channel fluorescence intensity (MFI) derived from fluorescence histogram was used to study the level of cell surface TLR expression. Delta MFI (dMFI) was calculated as a subtraction and recorded as the MFI of the TLR2 or TLR4 antibody minus the MFI of the isotype-matched control antibody.

### Genotyping of Toll-like receptor-2 and −4 genes

Genomic DNA was isolated from whole blood samples using ZR Genomic DNA II Kit (Zymo Research, Irvine, CA). Determination of the TLR 2 and 4 gene mutations was accomplished with polymerase chain reaction (PCR) and restriction fragment length polymorphism. Specifically designed PCR primers were used to amplify a region of the TLR2 and TLR4 gene spanning the respective single nucleotide polymorphism (SNP). The primer sequences are presented in Table 2.

**Table 2:** Sequences of primers used for PCR

**Gene and polymorphism**	**Primer 5**′**-3**′	**Annealing temperature [°C]**
TLR2 Arg677Trp	Forward: ccc ctt caa gtt gtg gct tca taa g	65
TLR2 Arg677Trp	Reverse: agt cca gtt cat act tgc acc ac	
TLR2 Arg753Gln	Forward: cat tcc cca gcg ctt ctg caa gct cc	65
TLR2 Arg753Gln	Reverse:: gga acc tag gac ttt atc gca gct c	
TLR4 Asp299Gly	Forward: agc ata ctt aga cta cta cct cca tg	62
TLR4 Asp299Gly	Reverse: aga aga ttt gag ttt caa tgt ggg	
TLR4 Thr399Ile	Forward: ggt tgc tgt tct caa agt gat ttt ggg aga a	60
TLR4 Thr399Ile	Reverse: gga aat cca gat gtt cta gtt gtt cta agc c	

The total volume of the PCR was 25 μl containing 50 ng of genomic DNA, 1x PCR-buffer (Qiagen, Hilden, Germany), 0.1 mM of each dNTP (Promega, Mannheim, Germany), 1 unit of HotStar-Taq® DNA polymerase (Qiagen) and 10 pmol of each primer (TIB MOLBIOL, Berlin, Germany). The final concentration of MgCl_2_ was 4 mM for Asp299Gly, 1.5 mM for Thr399Ile, and 3 mM for Arg753Gln and Arg677Trp. The PCR was executed with an initial denaturation step (94°C for 15 min), 35 cycles (94°C for 30 s, 62°C (Asp299Gly), 60°C (Thr399Ile), or 65°C (Arg677Trp and Arg753Gln) for 30 s, 72°C for 30 s), and a final extension step (72°C for 10 min).

The restriction assay contained 1x restriction buffer, 7.5 units of the respective restriction enzyme (New England Biolabs, Beverly, MD, USA) and 10 μl of the PCR product. It was incubated overnight at 37°C and 60°C respectively and analysed by electrophoresis on a 2.5% agarose gel. Full length PCR products were digested into the restriction fragments as listed in Table 3.

**Table 3:** Restriction enzymes and length of the restriction fragments

**Gene and polymorphism**	**Restriction enzyme**	**Restriction temperature [°C]**	**Fragment length [bp]**
TLR2 Arg677Trp	MwoI	60°C	Wild type (allele C): 130 bp + 22 bp
(rs unknown)			Arg677Trp (allele T): 152 bp
TLR2 Arg753Gln	MspI	37°C	Wild type (allele G): 104 bp + 25 bp
(rs5743708)			Arg753Gln (allele A): 129 bp
TLR4 Asp299Gly	NcoI	37°C	Wild type (allele A): 188 bp
(rs4986790)			Asp299Gly (allele G): 168 bp + 20 bp
TLR4 Thr399Ile	HinfI	37°C	Wild type (allele C): 124 bp
(rs4986791)			Thr399Ile (allele T): 98 bp + 26 bp

### Statistical analysis

Mann–Whitney *U* test was used to compare data of healthy controls and JIA patients, respectively SLE patients. Kruskal Wallis test was used to compare data of JIA subtypes. The allele frequencies were calculated from the numbers of genotypes observed. The significance of differences in the allele frequencies among patients and control subjects was determined by Fischer’s exact test. χ^2^ analysis was used to test for deviation of genotype distribution from Hardy–Weinberg equilibrium and for comparison of differences in genotype combinations among patients and control subjects. Only the values of p < 0.05 were considered to be statistically significant in all analyses. Statistical analysis was performed with commercial software (SPSS Statistics Software version 15.0; SPSS Inc.).

## Results

### TLR2- and TLR4-surface expression

We examined the presence of TLR2 and TLR4 proteins on peripheral CD14^+^ blood monocytes of 65 JIA patients [(19 patients with systemic JIA (3 children under flare), 28 patients with oligoarthritis (3 children under flare), 9 patients with seronegative polyarthritis (3 children under flare) and 11 patients with enthesitis related arthritis (3 children under flare)] and 9 SLE patients (1 patient under flare) as well as 67 healthy individuals by flow cytometry analysis.

**Figure 1:**
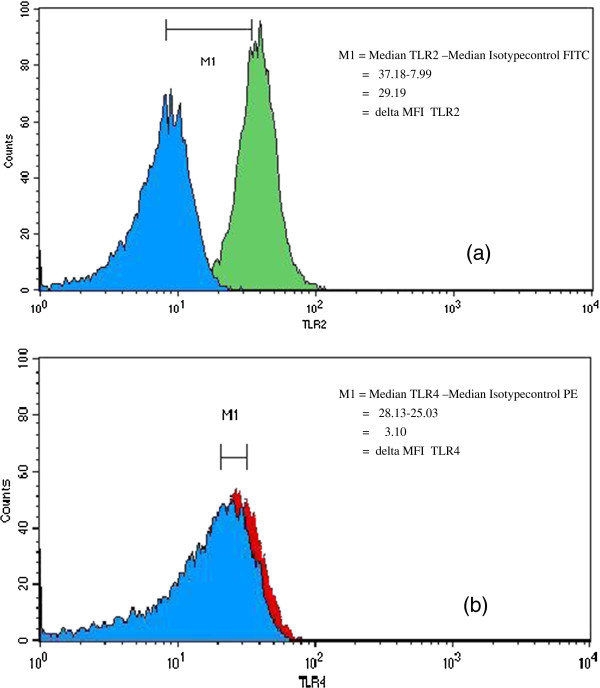
**Representative flow cytometry histograms showing (a) TLR2 and (b) TLR4 expression on CD14**^**+ **^**monocytes.** Blue filled histograms: isotype controls and green or red filled histogram TLR expression. Mean channel fluorescence intensity (MFI) derived from fluorescence histogram was used to study the level of cell surface TLR expression. Delta MFI (dMFI) was calculated as a subtraction and recorded as the MFI of the TLR2 or TLR4 antibody minus the MFI of the isotype-matched control antibody.

**Figure 2:**
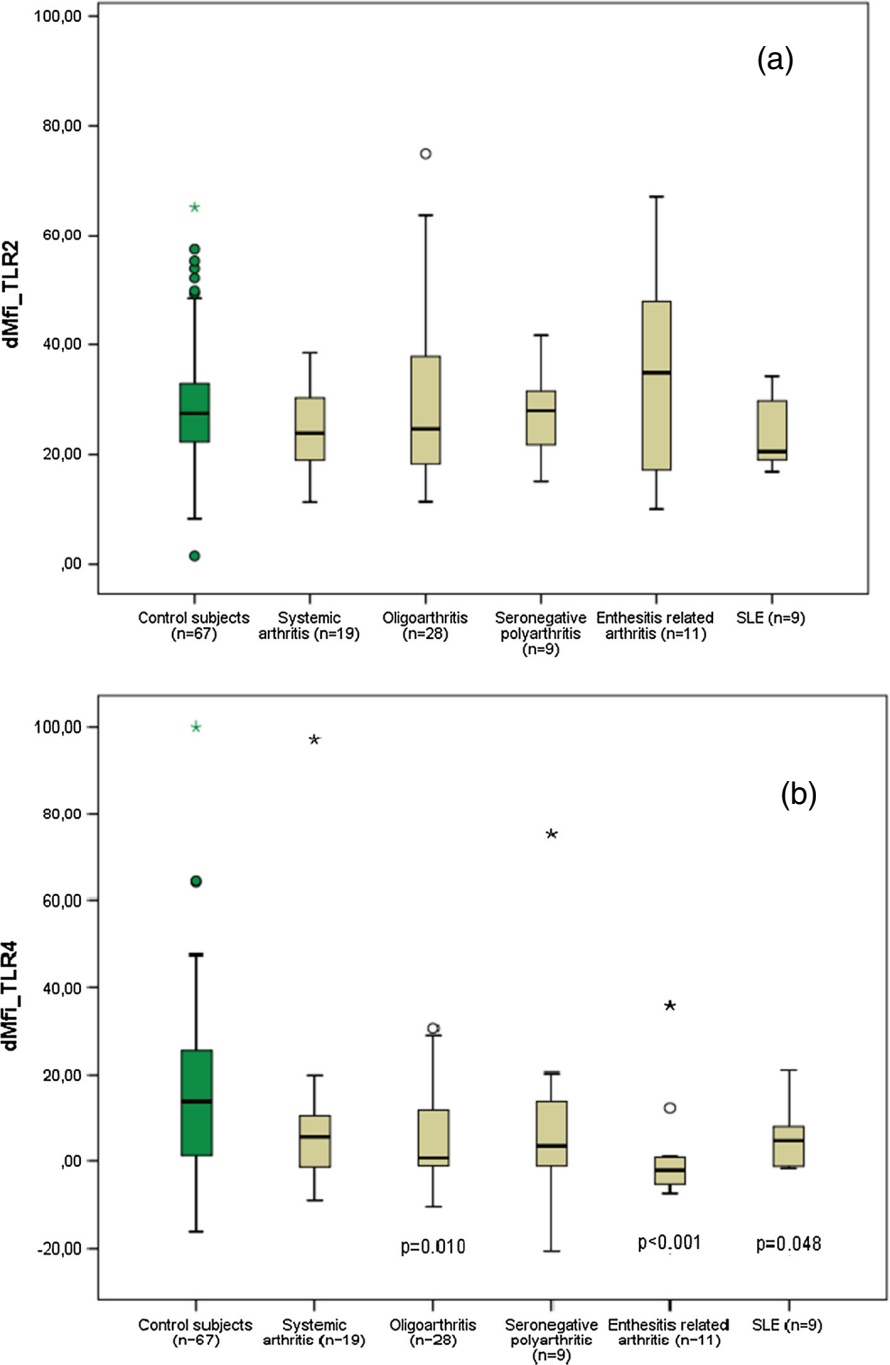
**(a) Mean fluorescence intensity (dMFI) of *TLR2-expression *on CD14**^**+ **^**monocytes of patients with JIA, SLE and healthy controls. (b)** Mean fluorescence intensity (dMFI) of *TLR4-expression* on CD14^+^ monocytes of patients with JIA, SLE and healthy controls.

We stated that monocytes expressed both TLR2 and TLR4 molecules, and expression of surface TLR2 was generally higher than TLR4 level (Figure 1). We observed no significant difference in the levels of TLR 2 expression, as measured by the dMFI, on the monocytes of JIA and SLE patients (median dMFI, 25.6, minimum dMFI 10, maximum dMFI 75) compared with the healthy control subjects (median dMFI 27.5, minimum dMFI 1.5, maximum dMFI 65.2; p = 0.239; Figure 2a).

**Figure 3:**
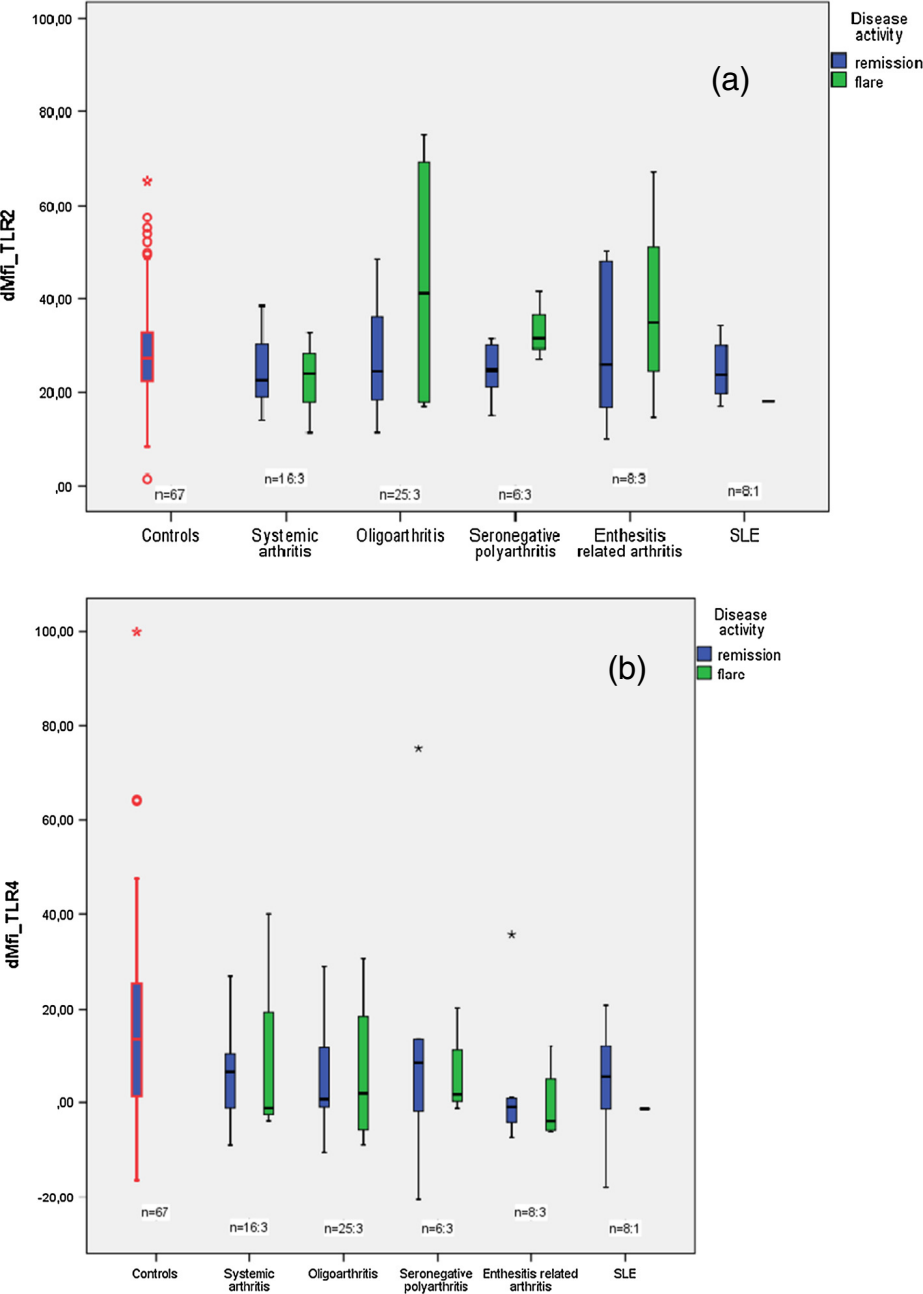
**(a) Mean fluorescence intensity (dMFI) of *TLR2-expression *on CD14**^**+ **^**monocytes of patients in various disease phases compared to TLR2-expression on monocytes of healthy controls. (b)** Mean fluorescence intensity (dMFI) of *TLR4-expression* on CD14^+^ monocytes of patients in various disease phases compared to TLR4-expression on monocytes of healthy controls.

By contrast, we found a significant reduction in the levels of TLR 4 expression on the monocytes of JIA and SLE patients (median dMFI, 1.3, minimum dMFI −20.6, maximum dMFI 97.1) compared with the healthy control subjects (median dMFI 13.5, minimum dMFI −16.4, maximum dMFI 99.9; p ≤ 0.001). Especially patients with enthesitis related arthritis (p ≤ 0.001), oligoarthritis (p = 0.010) and SLE (p = 0.048) showed significant reduction in the expression levels of TLR 4 compared with the control group (Figure 2b). Patients in acute flare showed no significant change in surface expression levels of TLR 2 (p = 0.299) and TLR 4 (p = 0.318) compared to patients in remission (Figure 3).

### Genotyping of Toll-like receptor-2 and −4 genes

**Figure 4:**
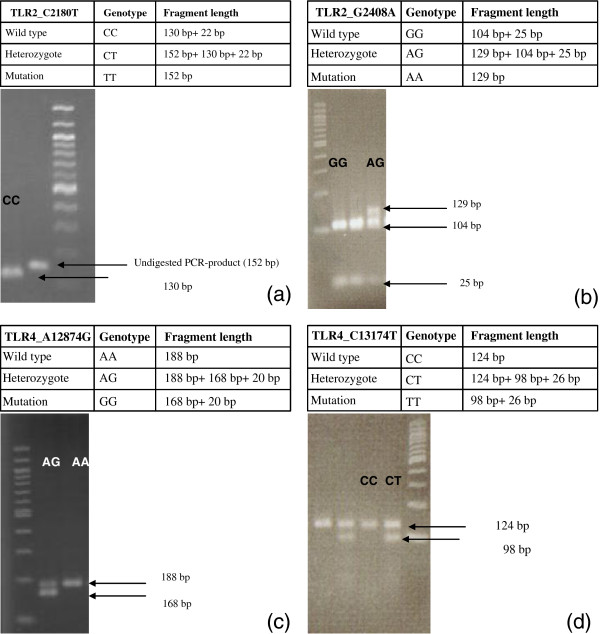
**Electrophoretic separation of TLR2 and TLR4 gene PCR fragments after restriction endonuclease cleavage on agarose gel (a-d): (a) Agarose gel electrophoresis of the TLR2 Arg677Trp polymorphism (TLR2_C2180T): lane 1, restriction fragments in case of 1 wildtype individual; lane 3, 100 bp DNA ladder; lane 2, PCR product without (before) restriction. (b)** Agarose gel electrophoresis of the TLR2 Arg753Gln polymorphism (TLR2_G2408A): lane 1, 100 bp DNA ladder; lanes 2 and 3, restriction fragments in case of two wildtype individuals; lane 4 individual heterozygous for the Arg753Gln mutation. **(c)** Agarose gel electrophoresis of the TLR4 Asp299Gly polymorphism (TLR4_A1287T): lane 1, 100 bp DNA ladder; lane 2 restriction fragments in case of an individual heterozygous for the Asp299Gly mutation; lane 3, restriction fragments in case of 1 wildtype individual. **(d)** Agarose gel electrophoresis of the TLR4 Thr399Ile polymorphism (TLR4_C13174T): lanes 1 and 3, restriction fragments in case of 2 wildtype individuals; lanes 2 and 4, restriction fragments in case of two individuals heterozygous for the Thr399Ile mutation; lane 5, 100 bp DNA ladder.

Determination of the TLR 2 (Arg677Trp and Arg753Gln) and 4 gene mutations (Asp299Gly and Thr399Ile) was accomplished with PCR and restriction fragment length polymorphism (Figure 4). In the present study compared the allele frequency and genotype distribution for these mutations among JIA respectively SLE patients and healthy control subjects.

Regarding the TLR 2 gene, the frequency of the Arg753Gln mutant allele was 1.3% (1/76) for patients and 9.0% (6/67) for healthy controls (p = 0.051). Except for one SLE patient, who was heterozygous for the Arg753Gln SNP, all the investigated subjects were wild-type for the TLR 2 gene. The Arg677Trp mutant allele was not found in any of the JIA/SLE patients or healthy control subjects. None of the study subjects or control group showed homozygosity for the Toll-*like* receptor-2 and TLR-4 mutant alleles. Within the group of JIA patients, the allele frequency of the Asp299Gly mutation was 6.0% (4/67), the one for the Thre399Ile mutation was 3.0% (2/67). In comparison, the one in the control group was 9.0% (6/67) for the Asp299Gly mutant allele (p = 0.516) and 12% (8/67) for the Thre399Ile mutant allele (p = 0.114). Cosegregation between the mutant alleles of the TLR 4 gene was found in 3.0% among JIA patients and in 9.0% of healthy control subjects. There was no correlation between the TLR 2 or TLR 4 genotypes and the demonstrated differential cell surface expression.

## Discussion

The stimulation of various TLRs via pathogen-associated molecular patterns (PAMPs) and damage-associated molecular patterns (DAMPs) is considered to be the basis for the emergence of individual autoimmune diseases such as rheumatoid arthritis [7,8]. The present study reveals selective alterations in the expression of Toll-*like* receptors 2 and 4 on CD14^+^ monocytes in the systemic circulation of patients with JIA and SLE compared to healthy control subjects. Detectable differences in receptor density could give further evidence of an involvement of TLRs in the pathogenesis of JIA and SLE.

In the course of this study we demonstrated a significantly reduced level of cell surface TLR 4 protein expression on CD14^+^ monocytes compared with that of healthy control subjects. This finding supports the proposed role of TLR activation by microbial products or their PAMPS respectively DAMPs in the pathogenesis of JIA and SLE: TLR stimulation via endogenous and exogenous ligands may induce the internalization of the respective TLR and may result in the downregulation of cell surface TLR expression [22-24]. Former studies on in vitro and in vivo LPS stimulation demonstrated downregulation of TLR 2 and TLR 4 cell surface expression on human peripheral blood neutrophils and monocytes [25-27].

In particular, the constantly reduced TLR 4 surface protein levels during active and inactive disease phases compared with healthy individuals are further evidence supporting a pathogenetic role of PAMPs, DAMPs and TLRs in the development of JIA or SLE. The stability of TLR surface expression in the investigated stages of the disease could be an expression of a permanent lowering of TLR 4, but need not necessarily be accompanied by a loss of receptor function [28].

In a recent study with fibroblast-*like* synovial cells, high levels of TLR 2 and TLR 4 messenger RNA (mRNA) were detected in JIA patients. Furthermore, the stimulatory effect of bacterial, viral and endogenous TLR ligands on the production of destructive acting cytokines IL-6, IL-8 and matrixmetalloproteinases-1 and −3 was confirmed for both groups of patients [29]. However, studies on TLR surface expression on monocytes from sepsis patients suggest that increased TLR-mRNA concentrations not necessarily with increased amounts of cell surface protein. Although LPS causes a heightened transcription of TLR 2- and TLR 4-messenger RNA in monocytes, only the TLR 2 surface expression but not the TLR 4 expression appears to correlate with the TLR-mRNA concentration [25,27].

The systemic circulation of JIA, RA and SLE patients likely abounds with endogenous and exogenous TLR2- and TLR4 ligands, leading to receptor activation and subsequent cytokine production. Cytokines, chemokines and enzymes can, in turn, affect the TLR activation. In particular, the proinflammatory cytokines IL-6 and TNF-α appear to have a LPS-like effect on the TLR4-expression of human monocytes. While TNF-α inhibits TLR-mRNA formation and the TLR 4-receptor expression, IL-6 causes the opposite effect [30]. Moreover the anti-inflammatory cytokine IL-10 can reduce the concentration of TLR4-mRNA in monocytes of healthy individuals [31].

From our former studies we know, that JIA patients show compared to healthy subjects significantly higher levels of proinflammatory cytokines. Furthermore, we observed higher concentrations of the anti-inflammatory cytokine IL-10 in patients with oligoarthritis, seronegative polyarthritis and enthesitis related arthritis compared to healthy subjects (unpublished data).

TLR2 and TLR4 genotypes of patients with JIA and SLE were chosen to investigate whether the different TLR surface protein expression levels in these patients were due to any of the reported functional polymorphisms in these TLR genes [20,32,33]. These polymorphisms have been associated as well with several autoinflammatory diseases, such as rheumatoid arthritis, as well as with the susceptibility to bacterial infections [34-36]. Especially the Asp299Gly SNP of TLR 4 has been shown to correlate positively with an endotoxin hyporesponsive phenotype in humans [20,32]. Interestingly, within the group of JIA patients the allele frequency of the Asp299Gly mutation was only 6.0% (4/67) compared with an allele frequency of 9.0% (6/67) within the control group (p = 0.516). The Arg753Gln polymorphism of the TLR2 gene also has been reported to be of pathogenetic significance [33]. However, except for one SLE patient, who was heterozygous for the Arg753Gln SNP, all the investigated subjects were wild-type for the TLR2 gene. This finding indicates that the observed reduced TLR4 protein expression on monocytes of JIA and SLE patients is not due to a known functional SNP in the TLR4 gene, but instead, it is supportive of a kind of tolerance to PAMPs and cellular danger signals. This tolerance may result from former activation of TLR4 signaling pathway by recent exposure to microbial or endogenous ligands. Hence, these results do not confirm a significant role of the described SNPs in the TLR genes in the observed differential TLR protein expression levels on monocytes from patients with JIA and SLE, compared with that in healthy individuals.

## Conclusions

To conclude, our observations suggest involvement of investigated TLRs in the pathogenesis of JIA and SLE. It still remains to be elucidated whether reduced TLR4 expression is cause of chronic arthritis or a result of some feedback loop.

Although the results of this study for surface expression of TLR2 and TLR4 within JIA and SLE patients differ, in some aspects, from the results of other studies, they do not necessarily contradict them. In our own experiments (unpublished data) we have shown that the technical implementation affects the density of TLRs on monocytes significantly: Unstimulated monocytes expressed TLR2, TLR4, and CD14 in whole blood with an identical pattern to that seen in purified peripheral blood mononuclear cells (PBMC), although with lower mean fluorescences than were seen in purified cells. After stimulation with Lipopolysaccharid (TLR4) or Zymosan (TLR2) in whole blood we observed the expected increase of TLR2 respectively TLR4 on monocytes. In contrast, in purified PBMC populations we found as a result of Zymosan stimulation a significant decrease of TLR2 density (dMFI unstimulated: median 100, dMFI stimulated: median −400) and an increase of TLR4 density (dMFI unstimulated: median 30, dMFI stimulated: median 600) on monocytes.

Additional studies of TLR-mRNA expression in monocytes of these patients could possibly confirm a negative correlation with respect to the amount of TLR4 protein and TLR4-mRNA concentration. Further experiments with TLR antagonists may help to evaluate the cytokine network activated by TLRs.

## Competing interests

The authors declare that they have no competing interests.

## Authors’ contributions

MK carried out the flow cytometry and the molecular genetic studies, collected and compiled statistics and drafted the manuscript. SS collected patient demographics. AS conducted medical examination of patients, collected patient demographics and developed scoring system for clinical status of patients. VNU developed scoring system for clinical status of patients. PS carried out parts of the flow cytometry. WM-L is project leader and planned the study. WM-L conducted medical examination of patients, collected patient demographics and developed scoring system for clinical status of patients. All authors read and approved the final manuscript.
